# Book Review: The Big Five in SLA

**DOI:** 10.3389/fpsyg.2021.710042

**Published:** 2021-06-14

**Authors:** Yuanyuan Miao

**Affiliations:** School of Foreign Studies, Guangzhou University of Chinese Medicine, Guangzhou, China

**Keywords:** second language acquisition, neuroticism extraversion, openness to experience, agreeableness, conscientiousness

The importance of learning individual differences (IDs) has been well-documented in the existing literature. Personality trait as one of these IDs has been the focus of attention in psychology, but it has received little attention in second language acquisition (SLA) studies. Consequently, there is a dire need for SLA researchers to come to the grips with scrutinizing personality traits from different vantage points. To bridge this gap, Ewa Piechurska-Kuciel's well-written monograph, entitled *The Big Five in SLA*, is an opportune volume that unpacks how the Big Five dimensions (Neuroticism Extraversion, Openness to Experience, Agreeableness, and Conscientiousness) and their subsequent six facets can be implemented theoretically and empirically in SLA studies.

This thought-provoking monograph comprises four chapters, the first two of which deal with psychology, whereas the other two consecutive chapters primarily pertain to SLA. Chapter 1 is divided into three sections. The first section presents a panoramic synopsis of personality studies and sketches the historical and theoretical conceptualizations and foundations of personality, focusing on humanistic, psychoanalytic, as well as viewpoints. The prime aim of the second section is to delineate two trends, namely type and trait theories, with a focus on measuring instruments. Whereas type theories, enlightened by Jung's (1923) and Myers's (1980) taxonomies, concentrate on qualitative diversities and discrete classifications, trait theories, conceptualized by Allport's (1961), Cattell (1980), and Eysenck's (1950) ramifications, focus on formulating the latent structure of personality on the basis of statistical analyses. The author aptly justifies that the trait model has made the theoretical underpinning of the Big Five. The third part is designated to an elaboration of the significant theories, scrutinizing the advancement of personality across a lifespan based on psychosexual, psychosocial, cognitive, and social-cognitive perspectives.

Chapter 2 is the lengthiest and most comprehensive chapter, which expounds on the analysis of the Big Five traits, their theoretical underpinnings, and their consequences. Informed by the Five-Factor Theory, Piechurska-Kuciel thoroughly explicates the detailed review of each personality trait, which lucidly refer to its higher-order meta-trait of Stability or Plasticity and lower-order phenotypic dimensions. Chapter 2 also contains a succinct explanation of the prevailing tools used to assess personality traits. To me, what seems genuinely informative in this chapter is the elaborate discussion on each of the five dimensions along with their six facets, which are subsumed under each dimension. Equally importantly, each dimension is scrutinized in terms of its most dominant cognitive and academic, socio-affective, and behavioral consequences which are vividly instantiated in the social and cognitive nature of language learning in both academic and naturalistic contexts. Finally, the chapter describes gender and age differences, which is followed by a synopsis of the advancement of the Big Five traits across the lifespan in language learning.

Highlighting the pivotal role of personality traits in SLA is the focus of Chapter 3. This chapter succinctly overviews studies in SLA, elaborates on the fundamental terminologies, presents the idiosyncratic nature of SLA, and outlines the typologies of individual learner differences. Furthermore, the chapter thoroughly appraises the theoretical propositions and empirical approaches for every dimension of the Big Five, with respect to behavioral, cognitive and academic, social, and affective ramifications.

Chapter 4 integrates the theoretical and empirical studies on personality traits in SLA, paving the way for avenues for future research. Moreover, it problematizes the inconclusive research findings on personality studies in SLA and outlines the pedagogical interventions to boost specific strengths of given traits to attain a more comprehensive picture of foreign language learning effects. The chapter concludes with heightening the significance of scrutinizing personality not only to improve the teaching expertise but also the common well-being.

This informative monograph benefits its readers. Firstly, it comprehensively reviews the theoretical postulations and empirical studies of personality traits with regard to the Big Five traits. Secondly, the integration of the Big Five traits within the SLA studies is another unique feature of this volume that sheds more light on our understanding of the critical role of personality traits in our EFL/ESL classes. The third merit of this book is that it scrutinizes each dimension of the Big Five with respect to higher-order meta-trait of Stability or Plasticity and lower-order phenotypic aspects that help teachers and learners know how to be well-socialized in the pursuit of their personal growth. However, had the author elaborated on how biological and genetic approaches can affect personality traits, how culture can influence the Big Five, and how our personality and identity can impact each other, it would have been more insightful. Moreover, the readers would have been engaged more interactively had the author included a schematic representation of the Big Five Model and all its six facets for each of its dimensions as shown in Figure 1.

**Figure 1:**
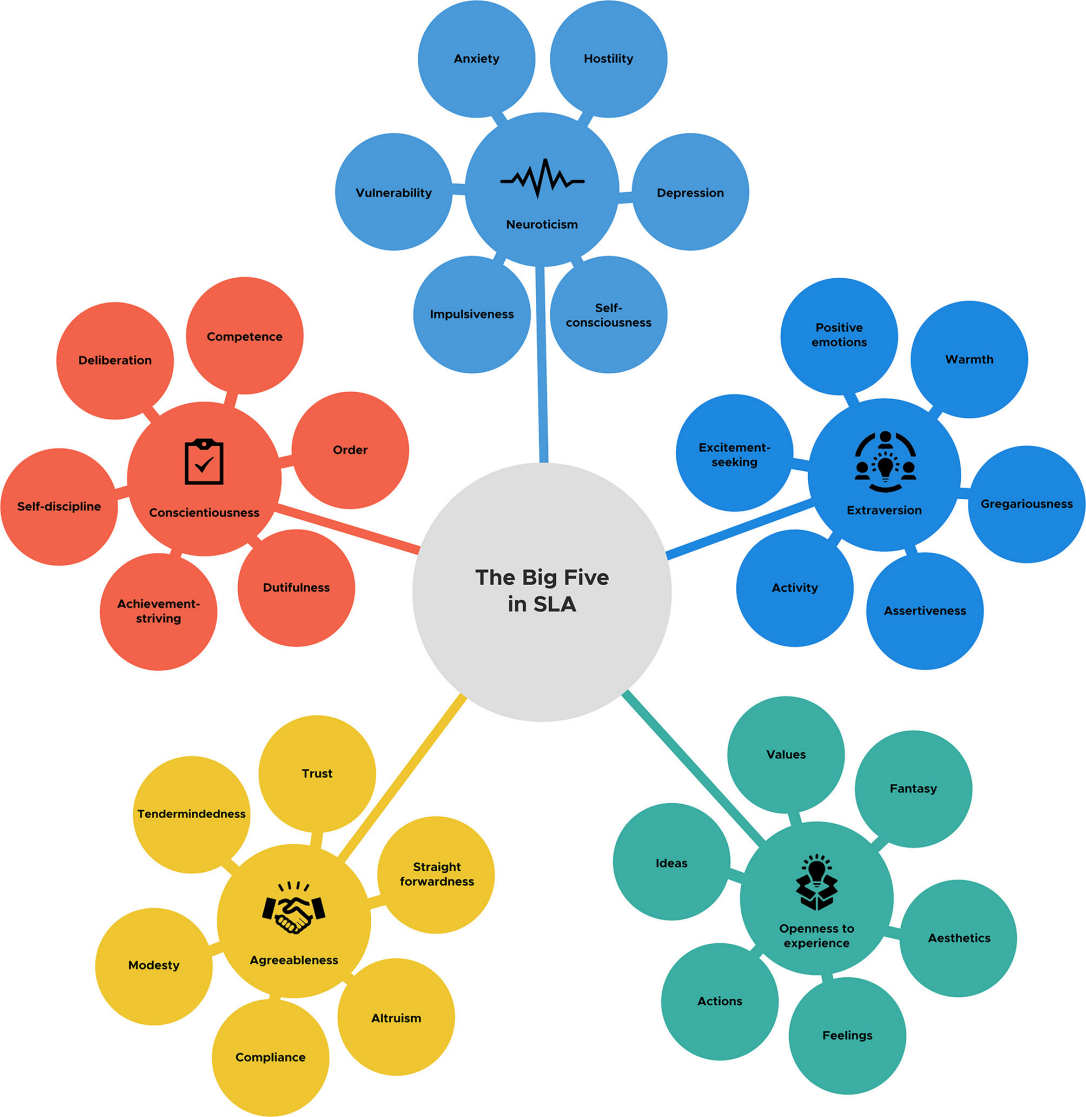
The schematic representation of the big five model and its six facets.

To conclude, this thought-provoking monograph provides plenty of food for thought for those who are enthusiastic about learning about the intricacies of personality traits in the context of SLA, including applied linguists, teachers, students, researchers, teacher educators, and syllabus designers. It is hoped that more empirical studies, using longitudinal and mixed-methods designs, are conducted on how the Big Five model can be practically applied in second and foreign language classrooms.

## Author Contributions

YM alone fulfilled design of the study, organized the database, and wrote all sections of the manuscript.

## Conflict of Interest

The author declares that the research was conducted in the absence of any commercial or financial relationships that could be construed as a potential conflict of interest.
