# Corrigendum: Book Review: The Big Five in SLA

**DOI:** 10.3389/fpsyg.2021.745119

**Published:** 2021-08-16

**Authors:** Yuanyuan Miao

**Affiliations:** School of Foreign Studies, Guangzhou University of Chinese Medicine, Guangzhou, China

**Keywords:** second language acquisition, neuroticism extraversion, openness to experience, agreeableness, conscientiousness

In the original article, a funder was neglected, **Guangzhou Social Science Planning Co Construction Project in 2021—Study on the Multi-Humanistic Path of the Overseas Spread of Traditional Chinese Medicine from the Perspective of Systematic Philosophy (No: 2021GZGJ97)**.

In the published article, there was an error in the affiliation. Instead of “**School of Foreign Studies, Guangzhou Medical University, Guangzhou, China**,” it should be “**School of Foreign Studies, Guangzhou University of Chinese Medicine, Guangzhou, China**.”

The author apologizes for this error and states that this does not change the scientific conclusions of the article in any way. The original article has been updated.

## Publisher's Note

All claims expressed in this article are solely those of the authors and do not necessarily represent those of their affiliated organizations, or those of the publisher, the editors and the reviewers. Any product that may be evaluated in this article, or claim that may be made by its manufacturer, is not guaranteed or endorsed by the publisher.

